# Inclusion of Beef Heart in Ground Beef Patties Alters Quality Characteristics and Consumer Acceptability as Assessed by the Application of Electronic Nose and Tongue Technology

**DOI:** 10.3390/foods13050811

**Published:** 2024-03-06

**Authors:** Savannah L. Douglas, Gabriela M. Bernardez-Morales, Brooks W. Nichols, Gabriella F. Johnson, Linda S. Barahona-Dominguez, Ainsley P. Jessup, Aeriel D. Belk, Jase J. Ball, Sungeun Cho, Jason T. Sawyer

**Affiliations:** 1 Department of Animal Sciences, Auburn University, Auburn, AL 36849, USA; sld0060@auburn.edu (S.L.D.); gzb0063@auburn.edu (G.M.B.-M.); bwn0004@auburn.edu (B.W.N.); gfj0006@auburn.edu (G.F.J.); adb0097@auburn.edu (A.D.B.); jase.ball@zoetis.com (J.J.B.); 2 Department of Poultry Sciences, Auburn University, Auburn, AL 36849, USA; lsb0042@auburn.edu (L.S.B.-D.); apj0027@auburn.edu (A.P.J.); szc0181@auburn.edu (S.C.)

**Keywords:** beef heart, cooked color, electronic nose, electronic tongue, ground beef patties, sensory analysis

## Abstract

Consumer purchasing of beef is often driven by the trinity of flavor, palatability, and convenience. Currently, beef patties in the United States are manufactured with fat and lean trimmings derived from skeletal muscles. A reduction in total beef supply may require the use of animal by-product utilization such as variety meats to achieve patty formulations. The current study aimed to assess textural, color, and flavor characteristics in addition to volatile compounds through electronic technology, e-nose and e-tongue, of ground beef patties formulated with beef heart. Ground beef patties were manufactured with 0%, 6%, 12%, or 18% beef heart, with the remainder of the meat block being shoulder clod-derived ground beef. Patties (*n* = 65/batch/treatment) within each batch (*n* = 3) with each treatment were randomly allocated to cooked color (*n* = 17/batch/treatment), Allo–Kramer shear force (AKSF; *n* = 17/batch/treatment), texture profile analysis (TPA; n = 6/batch/treatment), cooking loss (*n* = 17/batch/treatment), consumer panel (*n* = 3/batch/treatment), e-nose (*n* = 1/batch/treatment), and e-tongue (*n* = 1/batch/treatment) analysis groups. Patties containing beef heart did not require additional cooking time (*p* = 0.1325) nor exhibit greater cooking loss (*p* = 0.0803). Additionally, inclusion rates of beef heart increased hardness (*p* = 0.0030) and chewiness values (*p* = 0.0316) in TPA, were internally redder (*p* = 0.0001), and reduced overall liking by consumer panelists (*p* = 0.0367). Lastly, patties containing beef heart exhibited greater red-to-brown (*p* = 0.0003) and hue angle (*p* = 0.0001) values than control patties. The results suggest that beef heart inclusion does alter ground beef quality characteristics and consumer acceptability.

## 1. Introduction

Ground beef is a popular product purchased by meat consumers because of the meal versatility and affordability. Consumption patterns of ground beef in the United States from 2001 to 2018 comprised a minimum of 20 g daily for respondents ranging in age from 2 to 60 years old [1]. Beef is often the preferred red meat of consumers throughout the world and ranks third in per capita consumption of all meats globally [2]. It is well documented that consumer spending on meat proteins throughout the retail sector is price-driven as consumers are opting for less expensive cuts at the retail meat level [3]. Moreover, as economic conditions fluctuate each year, types of beef products purchased shift based on supply and demand [4]. Retailers have marketed ground beef as a product that can be selected for a lower purchase price than a whole muscle product such as a steak or a roast [5]. However, over the last decade, consumer demand has driven the need to improve the marketability of ground beef [5].

Advances in technology through applications of electronic multisensory instruments have been utilized in the food industry to assess sensory profiles like human senses of taste and smell [6,7,8]. However, limited efforts using e-tongue or e-nose technology have been focused on fresh or cooked ground meat samples such as beef. Previous efforts using rapid and non-destructive technologies have been focused on evaluating food spoilage and identifying toxic substances [9]. An electronic nose (e-nose) can be constructed with chemical gas sensors that measure volatile compounds or based on flash gas chromatography [10]. Technology such as the Alpha MOS e-nose uses ultra-fast gas chromatography, giving rapid and high throughput results [11]. The sensor array of an electronic nose can detect many components within a specified time frame and produce a specific response pattern [12]. Electronic nose techniques have been used sparingly to classify meat samples [13]. Using principal component analysis, reduced data sets have been able to discriminate between fresh and cooked characteristics of meat [12]. Technology such as the e-nose may be particularly useful in identifying potential pathogens that are harmful to human health and considered a food safety hazard without delays caused by conventional quantitative laboratory methods [12].

In addition, the electronic tongue (e-tongue) has been investigated as a method to discriminate and analyze sensory taste characteristics such as the five basic tastes of sweet, salty, sour, bitter, and umami [14]. A common method for taste detection in foods is to recruit and evaluate responses from trained descriptive or consumer sensory panels, but human sensory panelists cannot be used to evaluate food or drugs that are deemed harmful [14]. Moreover, human panelists often experience sensory taste fatigue or become difficult to recruit, which can alter study results. Therefore, the development of a sensor such as the e-tongue has proven capable in detecting basic taste properties correlated with chemical components, acids, and hydrogen ions [14]. Like the e-nose, analyzing samples with an e-tongue can aid in detecting freshness within foods and identifying many additives or adulterations of the product [14]. Using electronic technology in cooperation with human sensory panelists may provide a more wholistic interpretation of quality parameters for food products that have been previously limited.

Investigative efforts throughout the literature assessing the use of the electronic nose and tongue in beef, specifically ground beef, are limited. Early adoption of technology such as the e-nose was introduced as a method to evaluate the quality of fresh chicken meat [15]. Additionally, electronic analysis was used to identify meat sources from different breeds of beef cattle and domesticated buffalo [16,17]. It has been reported that the electronic tongue could identify the meat of different breeds with 100% accuracy [15]. However limited, the results published suggest that the methods of electronic analysis can be a successful monitoring step in the identification of meat and meat quality [15].

Consumers may not find the use of organ meats appealing; however, the use of animal by-product proteins such as beef heart in manufacturing ground beef may provide a nutritional benefit to consumers when beef inventories decline. Organ meats are some of the most nutrient-dense foods available and are naturally enriched with nutrients that are required for human survival [18]. Specifically, the heart is a source of copper, selenium, and iron, and it contains B12, but may contain excessive amounts of collagen [18]. The inclusion of variety meats in ground beef products could lower consumer product retail costs, provide an affordable value-added product, and improve environmental impacts by increasing the sustainable use of animal proteins for beef consumers. Adding organ meat can reduce lean trimming demands for the manufacturer and consumer cost [19].

Nevertheless, the use of beef organs such as the heart in ground beef has not been thoroughly studied; therefore, the objective of this study was to measure quality characteristics of ground beef patties with different inclusion rates of beef heart.

## 2. Materials and Methods

### 2.1. Raw Materials

Beef chuck eye rolls (USDA Institutional Meat Purchasing Specification #116A) and beef heart (USDA Institutional Meat Purchasing Specifications #1723) were purchased from a commercial meat distributor (Sherwood Foods, Atlanta, GA, USA) and transported to the Lambert Powell Meat Laboratory (Auburn, AL, USA) under refrigerated conditions (1.5 °C, ±1.0 °C). Raw materials were allocated to one of four treatments containing 0%, 6%, 12%, or 18% beef heart in 14.97 kg batches (three batches/treatment). Allocated raw materials were trimmed and coarsely ground once through a 9.525 mm plate (SPECO 400, Shiller Park, IL, USA) using a commercial meat grinder (Model AFMG-48, Biro Manufacturing Company, Marblehead, OH, USA). Each batch was mixed for 3 min, then ground once through a 3.18 mm plate (SPECO 400, Shiller Park, AL, USA) with a bone eliminator attached (SPECO 400, Shiller Park, IL, USA). Batches of ground beef were formed into 226.8 g patties (*n* = 65/batch/treatment) using an automated forming machine (Super 54, Hollymatic Corporation, Countryside, IL, USA). Formed patties were individually identified and vacuum packaged using 25.40 cm × 20.32 cm (l × w) 3 Mil vacuum pouches. A total of 780 patties were portioned, packaged, and frozen at −22.2 °C (±2.1 °C). Within each batch (*n* = 3), patties were assigned randomly to groups for laboratory analysis of cooked color (*n* = 17/treatment/batch), cooking loss (*n* = 17/treatment/batch), texture (*n* = 17/treatment/batch), electronic (e-nose/e-tongue) technology (*n* = 6/treatment/batch), and a consumer taste panel (*n* = 9/treatment/batch; Table 1). Packaged patties were stored at −22.2 °C (±2.1 °C) until laboratory analysis could be completed.

### 2.2. Proximate Analysis and pH Value

Beef patties (*n* = 1/batch/treatment) for determining relative proximate analysis (protein, moisture, and fat) were evaluated after packaging. Analysis was conducted using a near-infrared (NIR) spectrophotometer (Food Scan™, FOSS Analytical A/S, Hilleroed, Denmark), and data processing was determined using ISIscan™ Software (version 4.8, Höganäs, Sweden). Ultimate pH of ground beef was measured by weighing 2.00 g into a plastic centrifuge tube, adding 20 mL of deionized water, and homogenizing (Kinematica CH-6010, Brinkmann Instruments, Inc., Westbury, NY, USA) for 45 s. Homogenized ground beef pH was measured using a pH meter (Model-HI99163, Hanna Instruments, Woonsocket, RI, USA) equipped with a glass electrode. Calibration of the pH meter was completed (pH 4.0 and pH 7.0) using 2-point standard buffers (Thermo Fisher Scientific, Chelmsford, MA, USA) prior to sampling (Table 2).

### 2.3. Cooking Method, Cooking Time, and Cooking Loss

Before cooking, patties (*n* = 17/batch/treatment) were thawed for 12 h at 2.0 °C (±1.5 °C). A commercial cooking griddle (Model HEG36E-1, Vulcan, Baltimore, MD, USA) was pre-heated to 176.7 °C and each patty was cooked until reaching an internal temperature of 71.1 °C using a thermometer (Therma K-Plus, American Fork, UT, USA). Packaged patties were removed from packaging material after thawing and weighed on an analytical scale (Model PB3002-S, Mettler Toledo, Columbus, OH, USA). Throughout the cooking process, patties were turned every two minutes to minimize cooking variation, as described by the American Meat Science Association Meat Cookery Guidelines [20]. Cooking time was recorded as the time required to reach an internal temperature of 71.1 °C. After cooking, patties were cooled to room temperature and re-weighed on the calibrated scale to obtain final cooked patty weight. Cooking loss percentage was calculated by the following formula: [(cooked weight − raw weight) ÷ cooked weight × 100)].

### 2.4. Instrumental Cooked Color Measurement

Cooled, cooked patties (*n* = 17/batch/treatment) were sliced horizontally through the geometric center of the patty and scanned for internal cooked color using a HunterLab MiniScan EZ colorimeter (Model 45/0 LAV, Hunter Associates Laboratory Inc., Reston, WV, USA). Before the color measurement, the colorimeter was calibrated using a black and white tile per the manufacturer’s guidelines to ensure instrument accuracy. Instrumental color values were determined from the mean of three readings on the cooked internal surface of each ground beef patty using illuminant A, an aperture of 31.8 mm, and a 10° observer measuring lightness (L*), redness (a*), and yellowness (b*) of each ground beef sample [20]. In addition, relative values for hue angle were calculated using the following equation: tan^−1^ (b*/a*), with a greater value indicative of color shifting from red to yellow. Chroma (C*) was calculated as √a*^2^ + b*^2^, where a larger value indicates a more vivid color. Lastly, reflectance values from spectral range 400 to 700 nm were used to calculate color changes from red to brown (630 nm/580 nm).

### 2.5. Texture Analysis

Using a 5-blade Allo–Kramer attachment (AKSF) with a texture analyzer (Model TA-XT Icon, Texture Technologies Corp., New York, NY, USA), the objective tenderness of each patty was measured (*n* = 17/batch/treatment). Patties were cooked and cooled according to the same procedures described for measuring the cooked color. After cooling to room temperature (23.3 °C), with a load cell of 294.2 N and a speed of 3 mm/s, each patty was cut into a 6 × 9 cm^2^ sample. Each sample was sheared once, and the maximum peak force recorded during analysis was recorded as Newton (N) of shear force.

Texture profile analysis (TPA) was performed on each patty (*n* = 6/batch/treatment) at room temperature using a texture analyzer (Model TA-XT Icon, Texture Technologies Corp., New York, NY, USA). Six 1 × 1 cm^2^ samples from each patty were removed and subjected to a two-cycle compression test using a load cell of 294.2 N. Samples were compressed to 60% of their original height with a cylindrical probe (TA-25A) 50 mm in diameter with a cross-head speed of 6.00 cm/min. Texture profile parameters were calculated as hardness (kg), which is the force required to compress a sample; cohesiveness, which is the extent of sample deformation prior to rupture; springiness, which is the ability of a sample to return to its original shape after force is removed; and chewiness (kg × cm), which is the force needed to masticate a sample for swallowing (hardness × cohesiveness × springiness) according to previously described procedures [21,22,23].

### 2.6. Electronic Sense Testing

Objective sensory values were measured (*n* = 1/batch/treatment) with an electronic tongue (α-Astree II Electronic Tongue, Alpha MOS, Toulouse, France) designed to mimic human taste responses and record taste profiles. An electronic tongue contains seven sensors representing subjective values for sour, salty, umami, sweet, and bitter, and two sensors working as general purpose sensors according to previously described methods [23]. Water-soluble extraction (WSE) of the ground beef patty was obtained by mixing 20.00 g from each treatment with 100 mL distilled water, and homogenizing the mixture (Model 38BL54, Waring Products, Torrington, CT, USA) for 30 s. Homogenized mixture was centrifuged at 1500× *g* (Model D-37520 Osterode, Thermo Electron Corporation, Karlsrube, Germany) for 5 min at room temperature. Supernatant was removed and filtered by a 500 mL 0.45 μm PES filter unit (VWR International, LLC, Radnor, PA, USA) under vacuum (Welch Vacuum Technology, East Hanover, NJ, USA) to remove any excess solids. E-tongue sensors were placed into each sample for 120 s to record taste values. After each sample, deionized water was used as a cleaning solution for the e-tongue sensor for 10 s. Using the AlphaSoft (version 7.2.8, Toulouse, France) software, relevance indexes for captured volatiles were sorted from largest to smallest for statistical analysis.

Volatile compounds of raw patties (*n* = 1/batch/treatment) were analyzed by using an electronic nose (Heracles Neo e-nose, Alpha MOS, Toulouse, France) containing an autosampler. Compounds were tested using flash gas chromatography throughout the e-nose evaluation. Under a laboratory fume hood, 2.00 g of each patty was weighed and transferred into 20 mL e-nose vials. Vials were agitated at 500 rpm with a 50.0 °C incubation temperature for 20 min in the autosampler incubator to generate volatiles for headspace analysis. After incubation, the autosampler injector inserted 5000 mL of the headspace gas at 125 mL/s to concentrate the odor inside the trap. Trapping condition was maintained at 40.0 °C for 50 s. Hydrogen gas was used at a 1 mL/min flow rate to carry the volatile components into the non-polar (MXT-5) and polar (MXT-1701) capillary columns for chromatographic analysis using a parallel, two-flame ionization detector (FID1 and FID2). Both columns were 180 mm in diameter and 10 m long. Final temperature of the analysis sequence was increased to 250 °C at 1 °C/s temperature increments from the initial 40.0 °C temperature according to described methods [24]. Peaks of the chromatogram were identified by comparing the retention time of each compound with their corresponding retention indices.

### 2.7. Consumer Testing for Ground Beef Patties

Consumer panelists older than 18 years with a preference for consuming ground beef at least 3 to 4 times per month were recruited from the faculty, staff, and student body at Auburn University (Table 3). Prior to conducting consumer panel activities, Auburn University Institutional Review Board (IRB) approved the exempt use of human panelists for this study (Exempt Protocol Number 23-524 EX 2310). Treated ground beef patties with different inclusion rates of beef heart were evaluated by consumers (*n* = 96). Panelist serving order was computed using RedJade software (version 6.1, Pleasant Hill, CA, USA) so that panelists would evaluate random samples masked with 3-digit codes. Ground beef patties (*n* = 3/batch/treatment) were cooked to an internal temperature of 71.1 °C and cut into triangular samples (*n* = 4/patty) according to American Meat Science Association guidelines [20]. Cooked samples were presented randomly to the panelists in the isolated sensory booths under normal color-masked lighting. Panelists were instructed to use saltine crackers (Great Value Unsalted Tops, Walmart Inc., Bentonville, AR, USA) and room temperature water as a palate cleanser between each sample. Consumer panelists evaluated their first impression of “overall liking” of the samples on a 9-point hedonic scale (1 = dislike extremely; 9 = like extremely).

### 2.8. Statistical Analysis

Data were analyzed using the MIXED model procedures of SAS (version 9.2; SAS Inst., Cary, NC, USA) as a randomized complete block design. Random effects included batch and error, while treatment was the lone fixed effect. Least squares means were computed for all variables. Orthogonal contrasts were used to compare beef heart inclusion at any rate to the control (beef heart vs. no beef heart) in ground beef patties. When significant (*p* ≤ 0.05) F-values were observed, least squares means were separated using pairwise *t*-tests (PDIFF option).

## 3. Results and Discussion

### 3.1. Cooking Time

The cooking time of a product can be affected based on its size, water content, and cooking method [25]. The mean values for chemical composition presented in Table 2 are presented for reference only and for treatment definition. Rapid detection using NIR technology for chemical components suggests that the treatment formulations lacked wide ranges of variability among components of water, fat, and protein. Furthermore, the pH of patty formulations and cooking time for each treatment are presented in Table 4. There was a main effect of treatment on cooking time (*p* = 0.0017), with patties containing 6% or 18% added beef heart taking longer (*p* ≤ 0.0941) to reach an internal cooked temperature of 71.1 °C compared to patties containing 0 and 12% inclusion of beef heart. Griddle temperature, the moisture content of meat, and the thickness of the meat product may alter thermal properties when cooking meat proteins. It has been previously reported that ground beef patties containing an additional source of protein require longer cooking times than patties without added protein sources [26]. It is plausible that the added protein within the ground beef patties resulted in a greater concentration of moisture content within the protein fraction of the beef patty, therefore resulting in a greater need for heat for denaturation during the cooking period. However, the reduction in cooking time and cooking loss for beef patties containing 12% beef heart is unknown, but possibly linked to a slight increase in moisture and a reduced quantity of fat, as measured by NIR compositional analysis. Surprisingly, orthogonal contrast analysis of beef patties containing heart inclusion indicated that the cooking parameters of beef heart patties did not differ in terms of cooking time from control patties (*p* = 0.1325). Unfortunately, previous studies describing beef patty formulations with by-products such as beef heart are limited.

### 3.2. Cooking Loss

Cooking loss, which can often be overlooked, identifies the loss of product through the cooking process of thawing, dripping, or evaporative losses [27]. Greater cooking losses can result in large financial impacts on the food service industry [27]. Differences in cooking loss among patties are presented in Table 4. The main effect of treatment on cooking loss was impacted (*p* = 0.0001) by the inclusion of beef heart in ground beef patties. Ground beef patties formulated with 6% heart had greater cooking losses (*p* ≤ 0.0005) compared to patties formulated with 12 and 18%, but did not differ (*p* = 0.0725) from 0% control patties. Contrastingly, patties formulated with 18% beef heart exhibited similar cooking losses to the control (*p* = 0.0782) patties. Beef patties formulated with 12% beef heart had the least amount of cooking loss (*p* ≤ 0.0110) in comparison to the control and other inclusion rates. Orthogonal contrasts pooling all treatments with the inclusion of beef heart within patties exhibited no overall difference (*p* = 0.0803) in cooking loss when compared to control patties. It is well known that cooking losses can be attributed to several factors such as cooking duration, the thickness of the patties, moisture content, and even fat quantity. Cooking losses for beef patties in the current study align with foundational results for cooking losses associated with whole-muscle and ground beef products from the previous literature [28].

### 3.3. Texture Analysis

There was a main effect of treatment (*p* = 0.0001) on the objective tenderness of patties with varying inclusion rates of beef heart. Patties formulated with 6% beef heart had the greatest (*p* < 0.0001) AKSF compared to all other patty treatments (Table 5). Variations in objective tenderness suggest that tenderness may have been driven by cooking and moisture losses rather than beef heart inclusion. The current results for objective tenderness coincide with the increase in moisture loss and cooking time across treatments, as reported in formulation means (Table 2). Patties containing 18% beef heart had lower AKSF values in comparison to either control (*p* = 0.0055) patties or patties containing 6% (*p* < 0.0001) added beef heart. AKSF values for patties containing 12% and 18% beef heart were similar (*p* = 0.2955). Evaluating tenderness using an orthogonal contrast across all treatments indicates that there was no difference in objective tenderness values between patties with beef heart inclusion and control (*p* = 0.1801) patties. Object tenderness values can be correlated to sensory taste anchors of texture, and surprisingly, objective values tend to align with consumer panel ratings for texture. Consumer panelist ratings did not differ but numerically were greater for patties formulated with 6% beef heart. Changes in objective and subjective tenderness can be a result of greater connective tissue, increased moisture losses, a reduction in fat quantity, and even the cooking method. Previously, a study evaluating varying fat levels of ground beef reported that objective tenderness values did not differ between regular and lean samples; however, extra-lean (less fat) ground beef required more force to shear [29]. Retailer offerings of ground beef are often presented as regular, lean, and extra-lean based on the actual fat percentage. It is well documented that the quantity of fat within a meat product, especially ground beef, can have an indirect relationship with the tenderness of a product. As a meat product is cooked and the fat within the meat reaches a melting point, cooking losses will alter cooked yields and subsequently objective tenderness [30]. In addition, a greater shear force is correlated with a reduced fat content in a meat product [31]. Dissimilar to the current study, beef heart was used to create surimi and used in the formulation of frankfurters [32]. Frankfurters containing greater percentages of beef heart surimi were characterized as more tender by sensory taste panelists [32]. Additionally, this same study reported lower values of AKSF, and TPA hardness and cohesiveness for the frankfurters containing the greatest amount of heart surimi. Previous studies suggest that using heart as a source of raw materials can reduce objective tenderness values, which is supported by the current results [32].

Texture profile analysis (TPA) was conducted to measure the textural values of the beef patties (Table 5). Springiness did not differ regardless of treatment (*p* = 0.5974). Hardness of the beef patties is represented as the force necessary to achieve sample deformation [33]. Orthogonal contrasting reported that patties containing beef heart (6, 12, and 18%) were harder (*p* = 0.0030) compared to control beef patties (0%). Moreover, patties containing beef heart had a greater hardness value (*p* ≤ 0.0478) compared to the control. The previous literature evaluating texture profile analysis and Warner–Bratzler shear force suggests that both TPA and Warner–Bratzler tests provide different parameters for measuring objective tenderness [34]. TPA can be considered more useful in measuring the sensory texture of meat than the Warner–Bratzler method, but both objective measures provide a similar result [34]. Chewiness was numerically greater for patties containing 6% beef heart (*p* = 0.0408); however, there was no treatment main effect difference (*p* = 0.1721). Additionally, using an orthogonal contrast for beef heart treatments versus the control, chewiness increased with beef heart inclusion (*p* = 0.0316). A main effect of treatment was detected for cohesiveness (*p* = 0.0002), with 6% inclusion of beef heart having greater cohesiveness compared to all treatments (*p* ≤ 0.0234). Springiness is related to the recovery of the sample after the first cycle and in between the second cycle, as well as the viscoelastic properties [35]. The current results indicate no significant change throughout each treatment, summarizing that beef heart inclusion does not affect the springiness (*p* = 0.5974) of a sample, but it does provide better resilience of the product (*p* = 0.0002). Lastly, orthogonal contrasts pooling all three treatments for beef heart compared to control patties indicate no difference in springiness (*p* = 0.5291) or cohesiveness (*p* = 0.6379). These results suggest that heart inclusion can impact certain textural profile traits. Objective measures for beef patties provide further support that adding protein by-products may alter textural properties that can be detected by consumers.

### 3.4. Instrumental Color and Spectral Values

Cooked meat color is dependent on the denaturation stage of the myoglobin protein [36]. Denaturation of myoglobin occurs through heat, and the endpoint cooking temperature can alter the amount of denaturation along with the internal color of cooked patties. The previous literature suggests that raw material formulation used in the manufacturing phases of ground beef may contribute to altering the internal cooked color regardless of the endpoint temperature [26,36,37]. The current results (Table 6) suggest that patties containing beef heart were redder internally than control (0%) patties when looking at the treatment main effect (*p* = 0.0003). Meanwhile, an exploration involving the incorporation of value-added vegetable proteins in ground beef patties reported that the inclusion of vegetable proteins did not affect the cooked CIE L* values of ground beef patties [26]. In agreement with previous results, there was no significant difference (*p* = 0.2088), regardless of treatment, for lightness (L*) among beef patties. However, beef patties containing 6% and 12% beef heart had greater (*p* ≤ 0.0378) yellowness (b*) values than control patties or patties containing 18% beef heart. Nonetheless, lightness and yellowness values did not significantly differ in an orthogonal contrast of beef heart samples versus the control. However, indications of internal redness such as a*, red-to-brown color change, hue angle, and vividness were greater (*p* = 0.0014) for all patties containing beef heart compared to control patties, illustrating the impact beef heart inclusion has on cooked color values. Adding beef heart to the formulation of ground beef may have increased the total quantity of myoglobin, hence the greater objective results for redness values of a*, hue angle, and red-to-brown color change. Unfortunately, the total myoglobin of fresh and cooked patties was not measured. Additional research should elicit results confirming that the inclusion of beef heart may alter total myoglobin quantity when incorporated into a ground beef formulation.

### 3.5. Electronic Sense Testing

Electronic instruments such as the e-nose and e-tongue are used to evaluate the quality and sensory attributes of food. Rapid analysis instruments such as the e-tongue and e-nose have been designed to measure and mimic the sense of smell, taste, and vision [38]. Applications have included evaluating the wholistic quality and shelf-life of fresh meat in addition to adulteration detection [38]. The literature has reported that the electronic tongue can aid in determining shelf life, the wholesome quality of a product, and the detection of adulterated products [38,39]. Using various levels of chicken and pork mixed with mutton, the electronic tongue correctly identified samples containing various levels of adulterated chicken or pork [39]. The current results concluded that the e-tongue was able to detect differences among beef patties with or without beef heart within the formulation (Table 7). The e-tongue results represent the mean percent of relative standard deviation (%RSD) for each sensor. The mean values for the sensors within the current study ranged from 0.31% to 4.00%, and based on previous results, the document literature suggests that lower %RSD values are indicative of greater analytical precision [40]. Electronic tongue values for sourness increased (*p* = 0.0001) and were greatest for patties containing 12% and 18% beef heart, whereas saltiness was greater (*p* = 0.0086) for beef patties containing 12% and 18% beef heart. It is plausible that a greater concentration of beef heart inclusion may impart greater quantities of measured sodium within the beef patties when using electronic detection methods such as the e-tongue. The current results for sourness and saltiness are similar to a study where electronic tongue measurements of vegetable protein in ground beef patties increased [41]. Umami is often the one sensory anchor associated with beef products but difficult to detect using subjective measures. Umami values measured with the e-tongue were greater in patties containing 12% and 18% added beef heart (*p* = 0.0001) when compared to 6% and control beef patties. Moreover, treatment main effect values show significance in saltiness (*p* = 0.0086) and umami (*p* = 0.0001), suggesting the effect that beef heart inclusion has on samples. However, using orthogonal contrasts suggests that beef heart patties did not differ in saltiness (*p* = 0.4472), bitterness (*p* = 0.4932), or umami (*p* = 0.0748) compared to control patties. The e-tongue values suggest that the technology can distinguish differences among samples. However, the technology remains relatively new and iterations through development may lead to improved and quicker detection of meat quality parameters linked to subjective measures such as consumer and trained sensory evaluation. Additional research is needed to quantify the use of rapid analysis methods such as the e-tongue and the subsequent correlation with consumer taste panel responses.

Additionally, the electronic nose was explored to identify meat volatiles that are present within the beef patty treatments (Table 8). Electronic nose instruments are constructed to differentiate samples by measuring headspace volatiles. It has been documented that aldehydes and alcohols can be used to assess the oxidative state of a meat or food product or to identify the presence of microbes [42]. In the current study, 24 volatiles were repeated at least two times among sample replicates of beef patties. Volatile compounds have been reported within the literature to contribute to the aroma profile of meat and to ultimately influence flavor perceptions by sensory panelists [43]. Volatiles that are commonly related to meat include tridecanal, a distinctly beef-like aroma compound and constituent of species flavor, and 2,3-Octanedione, which is a result of lipid oxidation and creates a warmed-over flavor [44]. It has also been reported that another key volatile of cooked beef includes N-nonanal [45]. N-nonanal was present in all beef patties among all treatments within the current study, as expected for cooked beef. Additionally, volatiles butanol and propanal have been linked to dry-cured hams and subsequent lipid oxidation [46,47]. Overall, there was a variety of volatile compounds identified within the beef patties, confirming that the use of electronic technology such as the e-nose can be applied to measure compositional characteristics in beef products. Recorded volatiles within the patties that differed significantly represent volatiles that have been successfully measured within the documented literature [44,45,46,47]. The use of the electronic nose is promising for the meat and food industry as a rapid technique to evaluate food products. In a preliminary study, the electronic nose detected meat adulteration of uncooked and cooked ground beef with the inclusion of pork at various percentages [48]. It appears that technological developments such as the electronic nose may be used to separate or identify adulterated meat or food samples. However, additional testing of electronic and rapid methods for determining quality differences linked to sensory anchors should be conducted.

### 3.6. Consumer Panel

Subjective evaluation of sensory characteristics by consumer panelists was conducted to highlight hedonic ratings of beef patties formulated with added beef heart (Table 9). A main effect of treatment was detected (*p* = 0.0002) on overall liking by the consumer panelists. Consumer panelist ratings for overall liking were greater (*p* ≤ 0.0196) for beef patties with either 0% or 6% added beef heart compared to either 12% or 18% beef heart inclusion. Interestingly, consumer panelists identified that there were no differences (*p* = 0.0955) in the overall texture among patties across all treatments. Overall, there was not a significant difference in orthogonal contrasts for appearance (*p* = 0.4605), aroma (*p* = 0.4679), flavor (*p* = 0.2184), or texture (*p* = 0.3829), regardless of treatment. It appears that using orthogonal contrasts improved our understanding of the effects on inclusion of beef heart as consumer panelist ratings based on overall liking differed when patties contained beef heart. Consumer ratings and orthogonal contrasts suggest that consumer panelists could not accurately identify patties containing beef heart for anchors of appearance, aroma, flavor, or texture. Sensory taste and objective tenderness results suggest differences in beef patty formulations, but the detectable ranges vary. A previous study evaluating rice by-product as an ingredient in the formulation of chicken patties reported no difference in consumer preference between the control sample and the treatment that contained the least amount of rice bran [49]. Adding ingredients to a meat block may have limits that are easily detectable when the inclusion rate exceeds flavor and textural preferences of consumer panelists. Notably, there was a distinguishable difference in the characteristics of meat patties that had greater inclusion rates, as the values for consumer acceptance declined [49]. Furthermore, the current results agree with previous findings that consumer testing of taste and visual characteristics can be a determining factor in the success and acceptance of a new product [50].

## 4. Conclusions

Creating value-added beef patties using alternative proteins such as beef heart appears to show promise based on the current findings. The characteristics of meat quality are altered; it is estimated that 18% beef heart would reduce beef patty manufacturing costs by 10% per pound. Although the results suggest that consumer panelists can detect differences in sensory attributes, the patties containing 6% beef heart had the greatest numerical values in all sensory areas, suggesting that adding 6% or less of beef heart might be a viable option. The inclusion of beef heart exceeding 6% does alter the texture, cooked color, and sensory attributes of the beef patty, but this study could be a starting point for new product development. Technology such as the e-nose and e-tongue may provide new information regarding meat quality measurements, but additional testing and correlation with sensory taste and objective texture measurements are needed. Overall, with further research incorporating a variety of meats at a low inclusion percentage, this option could continue to prove viable as an alternative protein source for red meat consumers.

## Figures and Tables

**Table 1:** Treatment and patty allocation for ground beef patties with or without heart inclusion.

Treatment	Beef Chuck (%)	Beef Heart (%)	Batch ^1^ (kg)	Patties/Batch ^1^	Total/Treatment
1	100% Ground Chuck	0.00%	14.97	65	195
2	94% Ground Chuck	6.00%	14.97	65	195
3	88% Ground Chuck	12.00%	14.97	65	195
4	82% Ground Chuck	18.00%	14.97	65	195

^1^ Three batches per treatment were manufactured and patties allocated randomly within each batch for analysis.

**Table 2:** Relative mean values for proximate analysis and ultimate pH of beef patties with or without heart inclusion.

	0%	6%	12%	18%
pH	5.76	5.80	5.81	5.95
Moisture (%)	65.52 ± 1.52	65.49 ± 1.02	66.86 ± 0.69	65.04 ± 1.12
Protein (%)	23.80 ± 0.56	24.14 ± 0.85	24.47 ± 0.26	24.04 ± 0.68
Fat (%)	17.86 ± 2.06	17.52 ± 1.60	15.74 ± 0.68	18.61 ± 1.65

**Table 3:** Consumer sensory panel frequency demographics.

	Respondents	Percentage
**Sex**		
Male	40	42.10
Female	55	57.90
**Age**		
19 to 20 years	6	6.32
21 to 29 years	58	61.05
30 to 39 years	20	21.05
40 to 49 years	6	6.32
50 to 59 years	3	3.14
60 to 69 years	2	2.12
**Ethnicity**		
Caucasian	51	53.66
Latino/Hispanic	20	21.05
Asian or Pacific Islander	13	13.68
African American	7	7.37
Prefer not to respond	2	2.12
Other	2	2.12
**Income**		
Less than USD 30,000	72	75.79
USD 30,000 to USD 49,999	6	6.32
USD 50,000 to USD 79,999	8	8.42
Greater than USD 80,000	9	9.47
**Consumes Beef Patties**		
Once a day or more	2	2.10
More than 3 times per week	9	9.47
2 to 3 times per week	22	23.16
Once a week	22	23.16
2 to 3 times per month	24	25.26
Once a month	15	15.79
Less than once a month	1	1.06
**Purchases Beef Patties**		
Once a week	15	15.79
Once every 2 or 3 weeks	29	30.53
Once a month	28	29.47
Once every 2 or 3 months	14	14.75
Once every 4 to 6 months	5	5.26
Once or twice a year	2	2.10
Less than once a year	2	2.10

**Table 4:** Evaluation of cooking loss and cooking time on ground beef patties with or without heart inclusion.

	Treatment						
	0%	6%	12%	18%	SEM *	*p* Value	Contrast ^1^
Cooking Time (s)	713.58 ^b^	752.24 ^a^	702.69 ^b^	736.74 ^a^	10.122	0.0017	0.1325
Cooking Loss (%)	34.36 ^a,b^	35.06 ^a^	32.65 ^c^	33.66 ^b^	0.381	0.0001	0.0803

^a–c^ Mean values within a row lacking common superscripts differ (*p* < 0.05). * SEM, standard error of the mean. ^1^ Orthogonal contrast: beef heart vs. control.

**Table 5:** Objective tenderness values for beef patties with or without heart inclusion.

	Treatment						
	0%	6%	12%	18%	SEM *	*p* Value	Contrast ^1^
AKSF (N)	261.88 ^b^	312.75 ^a^	250.46 ^b,c^	243.68 ^c^	4.934	0.0001	0.1801
Hardness (kg)	4.45 ^b^	5.04 ^a^	5.44 ^a^	5.02 ^a^	0.216	0.0102	0.0030
Springiness ^2^	1.63	1.69	1.63	1.85	0.132	0.5974	0.5291
Cohesiveness ^3^	0.44 ^b^	0.48 ^a^	0.42 ^b,c^	0.41 ^c^	0.010	0.0002	0.6379
Chewiness ^4^	3.10	4.04	3.79	3.89	0.334	0.1721	0.0316
Reslience ^5^	0.208 ^a^	0.216 ^a^	0.187 ^b^	0.180 ^b^	0.006	0.0002	0.0467

^a–c^ Mean values within a row lacking common superscripts differ (*p* < 0.05). * SEM, standard error of the mean. ^1^ Orthogonal contrast: beef heart vs. control. ^2^ Values for springiness (ratio of the time duration of force input during the second compression to that during the first compression or length 2/length 1). ^3^ Values for cohesiveness (ratio of the positive force area during the second compression to that during the first compression, area 2/area 1). ^4^ Chewiness = hardness x cohesiveness x springiness. ^5^ Values for resilience (ratio of the time duration of force input to positive force input during the first compression or area 5/area 4).

**Table 6:** Instrumental cooked color of beef patties with or without heart inclusion.

	Treatment						
	0%	6%	12%	18%	SEM *	*p* Value	Contrast ^1^
L* ^2^	55.02	55.11	54.78	54.18	0.403	0.2088	0.3951
a* ^3^	15.86 ^b^	17.54 ^a^	18.59 ^a^	17.92 ^a^	0.478	0.0003	0.0001
b* ^4^	18.93 ^b^	19.55 ^a^	19.49 ^a^	18.95 ^b^	0.215	0.0217	0.0642
Chroma ^5^	50.41 ^a^	48.55 ^b^	46.85 ^c^	46.88 ^c^	0.651	0.0001	0.0001
Hue Angle (°) ^6^	24.61 ^b^	26.38 ^a^	27.04 ^a^	26.15 ^a^	0.438	0.0006	0.0001
RTB ^7^	1.84 ^b^	2.06 ^a^	2.22 ^a^	2.15 ^a^	0.077	0.0014	0.0003

^a–c^ Mean values within a row lacking common superscripts differ (*p* < 0.05). * SEM, standard error of the mean. ^1^ Orthogonal contrast: beef heart vs. control. ^2^ L*—values are a measure of darkness to lightness (a larger value indicates a lighter color); ^3^ a*—values are a measure of redness (a larger value indicates a redder color); and ^4^ b*—values are a measure of yellowness (a larger value indicates a more yellow color). ^5^ C* (Chroma) is a measure of total color (a larger number indicates a more vivid color). ^6^ Hue angle (°) represents the change in color from the true red axis (a larger number indicates a greater shift from red to yellow). ^7^ RTB is the reflectance ratio of 630 nm ÷ 580 nm and represents a change in the color from red to brown (a larger value indicates a redder color).

**Table 7:** Mean relative standard deviation (%RSD) of ground beef patties with or without heart inclusion measured by Alpha MOS e-tongue.

Sensors	Treatment						
	0%	6%	12%	18%	SEM *	*p* Value	Contrast ^1^
Sourness	0.59 ^c^	0.76 ^b^	1.03 ^a^	0.92 ^a^	0.455	0.0001	0.0001
General	1.78	1.78	1.92	1.93	0.093	0.4844	0.3552
Saltiness	1.63 ^b,c^	1.11 ^c^	2.32 ^a^	2.08 ^a,b^	0.234	0.0086	0.4472
Umami	1.85 ^b^	1.11 ^c^	3.28 ^a^	2.73 ^a^	0.239	0.0001	0.0748
Bitterness	0.32	0.47	0.28	0.44	0.083	0.3626	0.4932

^a–c^ Mean values within a row lacking common superscripts differ (*p* < 0.05). %RSD = (standard deviation ÷ χ) × 100. * SEM, standard error of the mean. ^1^ Orthogonal contrast: beef heart vs. control.

**Table 8:** Volatile compounds (relative area) measured using an electronic nose (e-nose).

	Treatment							
Group/Compound	0%	6%	12%	18%	Sensory Descriptors ^1^	SEM *	*p* Value	Contrast ^2^
***Alcohol***								
ethanol	2.17	2.17	2.30	2.68	Alcoholic	0.160	0.1017	0.4611
n-butanol	1.55	0.85	0.64	0.88	Fermented	0.224	0.0681	0.0681
heptan-2-ol	0.55	0.85	1.32	0.94	Fatty	0.134	0.2774	0.0131
2,3-Octanedione	1.37 ^a,b^	1.24 ^b^	1.57 ^a^	1.59 ^a^	Aldehydic	0.085	0.0165	0.3332
Glycerol	0.25	0.18	0.23	0.19	Almond	0.036	0.5654	0.3875
Butanethiol	0.27 ^a^	0.19 ^b^	0.14 ^c^	0.18 ^d^	Sulfurous	0.009	0.0005	0.0024
***Aldehyde***								
propanal	7.8	6.31	6.15	6.02	Etheral	0.911	0.4502	0.1171
n-nonanal	0.52 ^a,b^	0.48 ^a,b^	0.52 ^b^	0.54 ^a^	Tallowy	0.066	0.0103	0.9620
tridecanal	0.22 ^a^	0.19 ^a,b^	0.12 ^a^	0.21 ^a,b^	Aldehydic	0.181	0.0056	0.3308
3-Methlybutanal	2.23	2.09	1.64	1.11	Green	0.259	0.0631	0.1030
***Alkane***								
Hexane	4.37	2.70	2.50	2.03	Alkane	0.522	0.1946	0.0497
4-Methylheptane	0.4 ^a^	0.36 ^a,b^	0.28 ^a^	0.28 ^a^	-	0.082	0.0057	0.3710
***Ketone***								
Butane-2,3-dione	31.10 ^a^	18.46 ^b^	16.02 ^b^	32.21 ^a^	Butter	3.435	0.0294	0.0569
Acetoin	0.64	1.65	4.07	2.86	Sweet	1.003	0.1971	0.1902
Butan-2-1	28.16 ^a^	23.53 ^a,b^	21.34 ^a,b^	17.44 ^b^	Pungent	2.456	0.0195	0.0188
***Other***								
Carbon disulfide	76.31	85.56	87.7	148.82	Aromatic	18.565	0.0924	0.2025
Methyl butanoate	664.31	1327.87	1592.42	1590.85	Ester	177.055	0.0708	0.0708
Dibromochloromethane	26.66 ^b^	34.13 ^a^	42.49 ^a^	36.11 ^a^	Sweet	2.931	0.0326	0.0010
1,2—Benzenediol	0.25	0.21	0.17	0.18	Faint	0.032	0.4632	0.1202
1-Chloropentan	1.53	4.74	5.06	5.16	Green plant	0.460	0.0726	0.0343
E-2-Pentenal	0.48	0.37	0.34	0.39	Apple	0.036	0.0813	0.0126
1-Penten-3-1	0.62 ^a,b,c^	0.21 ^d^	0.65 ^b^	0.71 ^a^	Fishy	0.546	0.0013	0.2416
P-menthatriene	0.41	0.42	0.54	0.51	Woody	0.118	0.7902	0.5060
p-mentha-dien-hydroperoxide	83.23	82.96	82.95	83.18	Turpentine	0.128	0.3686	0.0001

^a–d^ Mean values within a row lacking common superscripts differ (*p* < 0.05). * SEM, standard error of the mean. ^1^ Sensory descriptor from AlphaSoft (version 7.2.8, Toulouse, France) Software Library. ^2^ Orthogonal contrast: beef heart vs. control.

**Table 9:** Consumer panelist ratings of ground beef patties with heart inclusion.

	Treatment					
Sensory Anchor ^1^	0%	6%	12%	18%	*p* Value	Contrast ^2^
Overall Liking	6.79 ^a^	6.89 ^a^	6.15 ^b^	6.37 ^b^	0.0002	0.0367
Appearance	7.04 ^a^	7.28 ^a^	6.59 ^b^	6.90 ^a,b^	0.0055	0.4605
Aroma	7.01 ^a^	7.19 ^a^	6.62 ^b^	6.89 ^a,b^	0.0254	0.4679
Flavor	6.47 ^a,b^	6.73 ^a^	5.86 ^c^	6.12 ^b,c^	0.0011	0.2184
Texture	6.78	6.96	6.4	6.42	0.0955	0.3829

^1^ Sensory anchor: consumer panelist ratings represent the use of a 9-point hedonic scale (1 = dislike extremely to 9 = like extremely). ^a–c^ Within a row, mean values lacking a common superscript differ (*p* < 0.05). ^2^ Beef heart vs. control.

## Data Availability

The original contributions presented in this study are included in this article, further inquiries can be directed to the corresponding author.
